# Pathway for Development and Validation of Multi-domain Endpoints for Amyloid Light Chain (AL) Amyloidosis

**DOI:** 10.1007/s43441-024-00641-6

**Published:** 2024-04-17

**Authors:** James Signorovitch, Jialu Zhang, David Brown, Preston Dunnmon, Liang Xiu, Nicolae Done, Kristen Hsu, Yolanda Barbachano, Isabelle Lousada

**Affiliations:** 1 Analysis Group, Boston, USA; 2 US Food and Drug Administration, Silver Spring, USA; 3 UK Medicines & Healthcare Products Regulatory Agency, London, UK; 4 Janssen Research & Development, Raritan, USA; 5 Amyloidosis Research Consortium, 320 Nevada Street, Suite 210, Newton, MA 02460 USA

**Keywords:** Clinical trial, Multi-domain endpoints, AL amyloidosis, Public–private partnership, Statistical validation, Rare diseases

## Abstract

Immunoglobin light chain (AL) amyloidosis is a rare disease in which a plasma cell dyscrasia leads to deposition of insoluble amyloid fibrils in multiple organs. To facilitate development of new therapies for this heterogenous disease, a public–private partnership was formed between the nonprofit Amyloidosis Research Consortium and the US Food and Drug Administration Center for Drug Evaluation and Research. In 2020, the Amyloidosis Forum launched an initiative to identify clinical trial endpoints and analytic strategies across affected organ systems and life impacts via specialized working groups. This review summarizes the proceedings of the Statistical Group and proposes a pathway for development and validation of multi-domain endpoints (MDEs) for potential use in AL amyloidosis clinical trials. Specifically, drawing on candidate domain-specific endpoints recommended by each organ-specific working group, different approaches to constructing MDEs were considered. Future studies were identified to assess the validity, meaningfulness and performance of MDEs through use of natural history and clinical trial data. Ultimately, for drug development, the context of use in a regulatory evaluation, the specific patient population, and the investigational therapeutic mechanism should drive selection of appropriate endpoints. MDEs for AL amyloidosis, once developed and validated, will provide important options for advancing patient-focused drug development in this multi-system disease.

## Introduction

### Immunoglobulin Light Chain Amyloidosis

**Figure 1:**
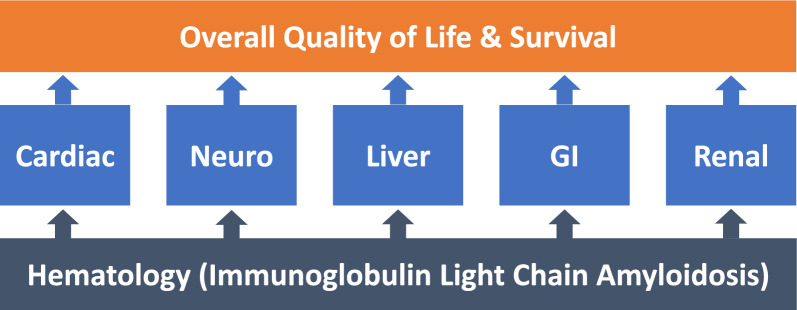
The underlying hematologic disease process in AL amyloidosis is plasma cell dyscrasia, which leads to deposition of insoluble amyloid fibrils in multiple organs, leading to multisystem clinical impacts that affect health-related quality of life and survival.

Immunoglobulin light chain (AL) amyloidosis is the most common type of systemic amyloidosis [1–4]. AL amyloidosis is caused by a monoclonal plasma cell disorder characterized by misfolding of light chains which leads to insoluble amyloid fibril deposits in target organs [1, 5] (Fig. 1). This rare, multi-systemic, and phenotypically heterogenous disorder affects cardiac, renal, neurological, gastrointestinal, and hepatic organ systems to varying degrees in different patients [1]. In patients with AL amyloidosis, outcomes are highly dependent on the spectrum and severity of organ involvement; cardiac involvement and/or progression to end stage renal disease are the primary causes of mortality [6, 7].

### Approaches to Develop Endpoints for Rare Multi-Systemic Disease

Given the multi-systemic nature of AL amyloidosis, disease modifying therapies would ideally have multi-domain benefits that are clinically meaningful for patients. A patient experience survey was conducted in more than 500 patients with AL amyloidosis involvement in the heart (36.7%), kidneys (28.1%), gastrointestinal tract (5.8%), nervous system (5.5%) or liver (3.2%) to better understand the burden of disease and attitudes toward available healthcare [8]. Across this broad spectrum of patients, nearly half had a positive view of clinical trial participation to enhance their clinical care. However, a broadly enrolled clinical trial representative of the heterogenous patient population with AL amyloidosis would likely confound the use of a single-domain primary efficacy endpoint other than mortality since some individuals will not be impaired at baseline in certain domains that are profoundly impaired for others.

Multi-systemic rare diseases, such as AL amyloidosis, pose unique challenges in drug development due to the limited population for evaluation and the inherent heterogenous complexity in the assessment of clinical benefit. Regulatory authorities, such as the US FDA, have recognized there are often appropriate circumstances for using different types of primary endpoints, including co-primary endpoints, multiple primary endpoints, composite endpoints, and multi-component endpoints [9] and have provided guidance for developing clinical outcome assessment (COA) based endpoints to enable regulatory decision making in the context of patient-focused drug development (PFDD) [10], including rare disease trials [11]. This series of methodological PFDD guidance documents was developed to inform on collection and submission of patient experience and other relevant data [12, 13] and to identify COAs considered important from the patient perspective [14].

As with other multi-systemic rare diseases, a composite multi-domain endpoint (MDE) for AL amyloidosis is an attractive approach to earlier assessment of efficacy in a broad and heterogeneous population. By combining signals for drug effects across domains, a composite endpoint may increase the power to detect efficacy for a disease modifying therapy that benefits multiple or all domains. An important risk to consider is whether the MDE also combines noise which can obscure an otherwise detectable drug effect on a single domain. Clinical interpretability of MDEs can also be more complex than single-domain endpoints. Therefore, MDEs must be carefully designed with input from clinical experts and patients and evaluated for well-defined contexts of use with appropriate data and methods. This summary of proceedings discusses strategic approaches in the construction and assessment of MDEs.

### The Amyloidosis Forum

The Amyloidosis Forum (https://amyloidosisforum.org) is a public–private partnership formed between the nonprofit Amyloidosis Research Consortium (ARC) and the FDA Center for Drug Evaluation and Research to identify scientific gaps that pose barriers to drug development for the treatment of AL amyloidosis [15]. The Amyloidosis Forum leverages patient perspective with expertise and resources in academia, industry, health outcomes research, and regulatory agencies in the precompetitive domain to support development of safe and efficacious therapies for patients with AL amyloidosis.

### Approach to Evaluation of Endpoints for AL Amyloidosis Trials

**Figure 2:**
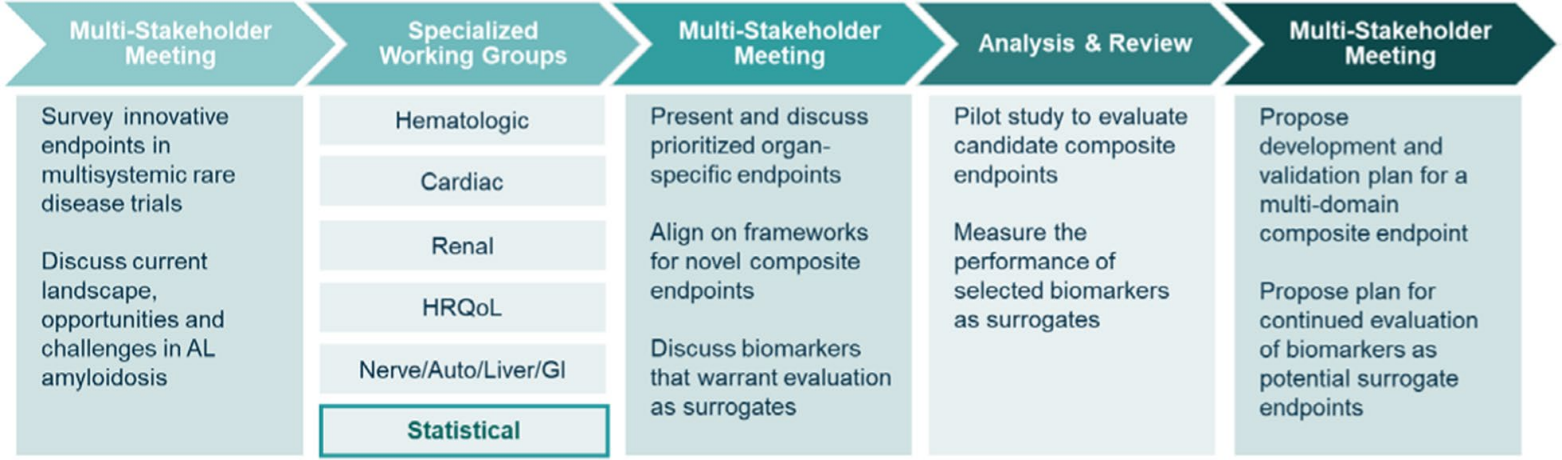
The Amyloidosis Forum set out to develop a novel multidomain composite endpoint and/or analyses methods for use in clinical trials for AL amyloidosis. Specialized working groups identified condition-specific and HRQoL outcome measures. The Statistical Group sought to identify and compare existing candidate MDEs previously used in clinical trials and practice deemed relevant across a broad spectrum of patients to facilitate drug development for the treatment of AL amyloidosis.

In 2020, the Amyloidosis Forum launched a series of virtual workshops to identify patient-relevant endpoint components and analytical strategies for clinical trials in AL amyloidosis (amyloidosisforum.org/novel-endpoint-and = analyses/). Specialized working groups in the areas of cardiac, hematologic, renal, and other (gastrointestinal, peripheral nerve/autonomic, hepatic) organ systems identified condition-specific endpoint components that could be used in clinical trials investigating novel therapies in AL amyloidosis (Fig. 2). Additional working groups reviewed health-related quality of life (HRQoL) measures and statistical approaches to analysis of clinical trial data. Proceedings of the Amyloidosis Forum are available at: https://amyloidosisforum.org.

This review summarizes the work conducted by the Statistical Group to propose a pathway for the development and evaluation of MDEs for AL amyloidosis clinical trials.

## Process for Endpoint Development

The Statistical Group was comprised of select members of the Forum, statisticians from the FDA and Medicines and Healthcare Products Regulatory Agency (MHRA), and a patient representative (IL). The chairperson (JS) led a series of meetings to assess MDE suitability for AL amyloidosis trials. The Statistical Group also reviewed publications and applications of MDEs in other therapeutic areas [16, 17] and consulted regulatory guidance from the FDA [9], European Medicines Agency [18], and the European Network for Health Technology Assessment [19] for the development and use of composite endpoints in drug evaluation. Each organ-specific working group presented available evidence and recommendations for appropriate endpoints for use in AL amyloidosis trials (Table 1). The Statistical Group considered several candidate MDE approaches along with recommended next steps to inform MDE development and to evaluate performance using natural history and available clinical trial data.

**Table 1:** Outcome Measures Prioritized by Amyloidosis Forum Working Groups

Organ System	Measure
Cardiac	Six-minute walk distance (6MWD) Kansas City Cardiomyopathy Questionnaire (KCCQ) Cardiac progression-free survival All-cause & cardiovascular (CV)-related hospitalization All-cause & CV-related mortality N-terminal pro-B-type natriuretic peptide (NT-proBNP) (for testing as a potential surrogate)
Nervous	Modified Neuropathy Impairment Score (mNIS + 7)^a^ Norfolk Quality of Life Diabetic Neuropathy (QoL-DN)^a^ Composite Autonomic Symptom Score (COMPASS-31) Rasch-built Overall Disability Scale (R-ODS) Neurofilament Light Chain^a^
Hepatic	Alkaline Phosphatase (ALP)^a^ (as a possible supportive biomarker)
Gastrointestinal	Patient-Reported Outcomes Measurement Information System Gastrointestinal Symptom Scales (PROMIS-GI)^a^
Renal	Proteinuria Estimated Glomerular Filtration Ratio (eGFR)

^a^No clinical thresholds yet validated in AL amyloidosis.

Summary based on presentations from Amyloidosis Forum Meeting: “Considerations for Novel Endpoint Development in AL Amyloidosis” (22 January 2021; available at: https://amyloidosisforum.org/considerations-for-novel-endpoint-development-in-al-amyloidosis/).

### Types of Multi-domain Composite Endpoints and Statistical Methodology

Different types of MDEs used in drug development across therapeutic areas were considered in terms of advantages and disadvantages for AL amyloidosis (Table 2). Additional approaches to develop a MDE may also be viable based on the mechanism of action of the intervention, target indication, study population, and trial design.

**Table 2:** Types of Composite Endpoints

Type of Composite Endpoint	Examples	Directly Sensitive to Delayed Worsening from Baseline	Directly Sensitive to Improvements from Baseline	Regulatory Precedent
Response Criteria	• ACR/EULAR (rheumatology) [20] • Hematological response [21] • Organ response [22]	No	Yes	Multiple full approvals
Time to a Composite Event	• Progression-free survival (PFS) in oncology trials [23] • Time to major adverse cardiovascular events (MACE) [24] • Major organ deterioration progression-free survival (MOD-PFS) [25]	Yes	No	Multiple full approvals
Finkelstein-Schoenfeld	• ATTR-ACT (survival, hospitalization) [26]	Yes	Possible	Recent full approvals
Multi-domain Responder Index	• Lysosomal storage disorders [27]	Yes	Yes	Post-hoc or exploratory

#### Composite Responder Criteria

Composite responder criteria, such as the American College of Rheumatology/European League Against Rheumatism (ACR/EULAR) classification criteria for rheumatoid arthritis [20], combine biomarkers and clinical outcomes important for near-term relief and longer-term disease control. Response is defined at the patient level as reaching or exceeding a given degree of improvement from baseline across the included domains [20, 28]. The timing of response assessment should be justified, based on the underlying disease biology and considering the multiple included domains, along with specificity on whether responder criteria must be satisfied *at* a specific time point or *at any time up to and including* that time point. Specificity is particularly important when underlying measures (e.g. NT-proBNP in AL amyloidosis) fluctuate over time. A response based MDE can be sensitive to drug effects on near-term improvements, but less sensitive to drug effects that slow or stabilize disease progression without improvement. A response based MDE offers easily interpretable results if the responder status is well established with clear clinical meaning. Caution should be applied for response outcomes that are derived by dichotomizing a continuous or ordinal measure. Such dichotomizations can complicate the interpretation of the overall treatment effect and may not be best practice. In some situations, a large responder effect may not necessarily reflect a clinically important treatment effect in a continuous or ordinal measure [19].

#### Composite Progression Endpoints

Composite progression endpoints have established precedent in cardiology, hematology, and oncology; progression-free survival (PFS) is a widely used example. In AL amyloidosis, the composite of major-organ deterioration progression-free survival (MOD-PFS), a secondary endpoint in the ANDROMEDA trial, supported accelerated FDA approval of daratumumab when added to bortezomib, cyclophosphamide, and dexamethasone (VCD) in patients with newly diagnosed AL amyloidosis [25, 29]. MOD-PFS is comprised of clinical endpoints including death, clinical manifestation of cardiac failure (defined as need for cardiac transplant, left ventricular assist device, or intra-aortic balloon pump), clinical manifestation of renal failure (defined as development of end stage renal disease evidenced by need for hemodialysis or renal transplant), or development of hematologic progressive disease per consensus guidelines [25]. Composite progression endpoints are often studied as the time to a subject’s first event, but recurrent events may also be applicable. Progression endpoints are directly sensitive to drug effects that slow the rate of progression, and indirectly sensitive to improvements persistent enough to delay progression. Composite progression endpoints are widely used and accepted in the regulatory setting when the progression criteria are clinically meaningful.

#### Hierarchical Composite Endpoints

The Finkelstein-Schoenfeld (FS) test [30] and the win ratio test [31] provide additional options for analyzing composite endpoints in the context of a randomized controlled trial design. Such tests are based on comparisons between all possible pairs of treated and untreated individuals. For each pair, the question is asked: who did ‘better,’ the treated or untreated individual? The individual with the better outcome is identified using a pre-defined hierarchy across multiple domains and can accommodate outcomes on different scales/types (e.g., time to event, categorical outcomes, continuous outcomes). Finkelstein and Schoenfeld first proposed a test statistic by performing pairwise comparisons on longitudinal and survival outcomes of all patients hierarchically. The FS test was used as the primary efficacy analysis in the ATTR-ACT trial of tafamidis in patients with transthyretin amyloid cardiomyopathy [32]. The approach works best when the component in the higher hierarchical order has a large treatment effect and a high event rate. The methodology can give priority to the more clinically important events and can also combine different types of components (longitudinal, outcome, categorical). However, caution needs to be taken when continuous measurements are included as components in the hierarchical composite endpoint. In such situations, the win-lose algorithm can be driven by a trivial difference from the continuous measurement and obscure the clinical meaning of the composite endpoint. The algorithm is computationally intensive, and the handling of censoring and missing data can be complicated. Other variants, generalizations, and approaches to combining multiple domains via statistical analysis have been proposed [33].

#### Multi-domain Responder Index (MDRI)

The MDRI encompasses multiple endpoints via pre-specified responder thresholds for each. Scores are assigned based on the outcome in each domain, then summed across all domains. For example, a score of + 1, 0, or −1 may be assigned for clinically significant improvement, no significant change, or clinically significant worsening, respectively, to an individual’s outcome in each domain. The total score summed across all domains represents the net number of domains improved vs. worsened for an individual, which can then be averaged and compared across treatment groups [34, 35]. MDRI was used in a post-hoc analysis of data from the pivotal trial of laronidase for the treatment of mucopolysaccharidosis I [36]. By design, MDRIs are sensitive to treatment effects that lead to improvement and/or the prevention of worsening across domains. In practice, MDRI can be difficult to implement and has not been accepted for pivotal trials in the regulatory setting. The responder threshold for each component should be clinically meaningful, which can be challenging to determine.

#### Composite Statistical Tests

There are also approaches that combine across component endpoints into a composite test. For example, the O’Brien Rank-Sum test considers the ranks of each outcome across both domains and patients, and assesses whether outcomes in the treatment group tend to be better ranked than those in the control group [37]. The Wei-Johnson test combines mean treatment effects across endpoints, including time-to-event endpoints, by weighting the contribution of each domain based on variance and correlation to produce a composite test [38]. These composite tests may also be viable approaches and should be further explored.

### Pathway for Development and Evaluation of Composite Endpoints for Use in AL Amyloidosis Trials

#### Identification of Principal Organ Systems, Domains, and Candidate Component Endpoints

The first step in development of a composite endpoint for use in AL Amyloidosis trials (or for any rare therapeutic indication characterized by multi-systemic involvement) involves identification of domains most indicative of disease progression and of disease aspects that patients consider most important for demonstration of treatment benefit (Fig. 1). Initially, the organ system-specific Working Groups reviewed available literature to identify available published reports on candidate component endpoint validation. If deemed insufficient, the Working Groups utilized clinical expert input and input from patients [39–41]. Considerations for each candidate endpoint included: (1) clinical relevance, (2) available natural history data and/or clinical experience, (3) time horizon to detect change based on natural history and timing of impact of effective treatment, (4) meaningful thresholds, and (5) gaps in knowledge.

#### Ensuring a Foundation for Clinical Meaningfulness

For an MDE to be considered clinically meaningful, each component of the MDE should be either:

- A *clinical endpoint* that measures how a patient feels, functions, or survives; or
- A *validated surrogate endpoint* predictive of treatment effects on how a patient feels, functions or survives

Surrogate endpoints must be validated separately; predictive associations between a potential surrogate and clinical outcomes are highly supportive, but not sufficient. Associations between treatment effects on surrogate response and treatment effects on overall survival, or on other clinical outcomes, should be established across multiple randomized trials [42–44]. Within-trial estimates of patient-level associations and proportions of the treatment effect on the clinical outcome explained by effects on the surrogate can complement cross-trial analyses [45].

The Prentice criteria provides a helpful framework for assessing surrogacy though a combination of statistical assessments and clinical/biological judgement about the pathways by which treatment might affect the surrogate and the ultimate clinical outcome [46]. Subsequent work has further extended and modified these criteria and emphasized the risks of ill-formed or un-validated surrogates [43, 44, 47, 48]. The approach of Buyse et al. [49] and its extensions has provided valuable empirical evidence of surrogacy supportive of validation alongside clinical and biological plausibility.

The FDA’s PFDD guidance provides recommendations on how to develop and validate COA based endpoints that reflect patients’ experiences and priorities and how to determine the clinical meaningful change in a COA based endpoint [10]. The interpretability of COA endpoints depends on how closely the measurement reflects patients’ experience and the COA metric used. To find the meaningfulness of a treatment effect in a COA measure, one can consider anchor-based methods by mapping an anchor, which is some external variable with direct interpretable difference (e.g., a patient or physician global assessment), to differences of the COA scores. Multiple anchors can be used to inform a plausible range of meaningful score difference (MSD).

The organ-specific working groups identified several candidates of high interest for constructing the MDEs, e.g. NT-proBNP, 6MWD, eGFR, and hematologic responses (Table 1). The applicability of these endpoints in AL amyloidosis needs further investigation and validation. For AL amyloidosis, understanding the potential surrogacy of NT-proBNP and hematologic response or progression outcomes is a priority. While changes in eGFR (including slope) have been accepted as a clinical endpoint in other therapeutic areas, applicability in AL amyloidosis needs further validation. With sufficient data from multiple randomized trials now available, research is underway within the Forum to conduct these assessments.

These steps provide a necessary foundation for identifying meaningful components of a composite MDE but are not alone sufficient for determining the clinical meaningfulness of the composite. For a composite, the method for combining information across domains also needs to be assessed for clinical meaning. Each individual component in a composite endpoint should be clearly understood in the analytic methods. When a composite endpoint shows a treatment effect, it’s necessary to also examine the treatment effect of each individual component.

#### Evaluating Component Endpoints

The evaluation of candidate endpoints for use as components has been discussed at public workshops [50]. Evaluation should assess clinical meaningfulness and reliability, as well as natural history in terms of rates of change and levels of variability over time in relevant patient populations and the degree of correlation among component endpoints.

Broadly, assessments of natural history provide drug developers with a quantitative understanding of component endpoint performance needed to evaluate suitability for incorporation into a composite clinical trial endpoint. Natural history of each component may be established using retrospective data from natural history studies/registries, post-hoc analysis of placebo data from completed clinical trials, or prospectively though pilot studies or during early phases of a clinical development program. Such assessments of endpoint components provide a foundation for regulators and other decision-makers to assess suitability, adequately power a trial, and to interpret trial results based on the endpoint composite.

The characteristics of candidate endpoints (e.g., 6MWD, eGFR, NT-proBNP, and neurological measures) should be further evaluated, including the rates of change and intra-subject variability. For potential surrogate endpoints, in-depth understanding of the causal pathway of the disease process would be ideal; as such, an extensive overview on available studies with reliable estimates on both the clinical outcomes and the biomarkers is recommended. For proposed response or progression thresholds, rates of response and progression—and rates of reversal of response or progression—should be quantified. Such data are necessary in making evidence-based choices for the combination of clinical trial enrollment criteria, endpoint selection, and trial follow-up duration to detect meaningful effects in the development of investigational products.

#### Development of Candidate Composite Multi-domain Endpoints

The Statistical Group discussed four ways of constructing multi-domain composite endpoints: (1) a response criterion (similar to the ACR); (2) time to a composite progression event (e.g., MOD-PFS); (3) an MDRI, and (4) a multi-domain hierarchical composite endpoint.

To inform the statistical performance and interpretation of composite MDEs, overlap across domains should be assessed both statistically and based on clinical judgement. If two component endpoints are very highly correlated and represent similar clinical concepts, this could unintentionally lead to effective “double counting” of the underlying domains. When reporting treatment effects on MDE outcomes, the treatment effect of each component should be reported in parallel to support interpretation [18].

In addition to establishing the clinical meaning of each component, it is necessary to characterize the clinical meaning of the composite MDE, particularly in the context of AL amyloidosis where different organ systems may be affected to varying degrees in different patients. Composite MDE outcomes may be quantified and qualified using natural history or placebo arm data in populations that could be enrolled in clinical trials. In particular, response rates and times to progression—and the stability of response or progression status—need to be assessed across different sets of inclusion/exclusion criteria. MDEs may combine different types of component endpoints, e.g., physician-assessed outcome assessments, patient-reported outcome assessments, performance outcomes, or drug-induced changes in biomarkers obtained from laboratory assays or imaging. When combining several types of components, it is not recommended to combine components with drastically different clinical importance. Such MDEs would often be challenging to interpret and determine clinical meaningfulness.

Ultimately, the performance of an MDE for AL amyloidosis clinical trials must be evaluated within the context of its intended use, including the specific population (e.g., heterogenous organ involvement), hypothesized multi-domain treatment effect (i.e., for a plasma-cell or amyloid-directed therapy), and the regulatory decision/claim being sought. The pathway proposed above will provide a foundation for endpoint selection and development of composite MDEs in AL amyloidosis. Specifically, clinical trial simulations, and power and sample size calculations will rely on evidence derived from natural history data. While pre-existing evidence and the pathway to develop composite MDEs can provide a strong foundation for endpoint selection in AL amyloidosis, there may be value in updating and tailoring this approach for specific drug development goals, e.g., for drugs with novel mechanisms of action, for populations defined by novel biomarkers, or to adapt to future changes in the standard of care. For these reasons, availability of up-to-date and high-quality natural history data is important for drug development in AL amyloidosis.

## Discussion

Given the complexity and multisystemic nature of AL amyloidosis, the Amyloidosis Forum initiated an effort toward identifying appropriate endpoints and analytical methodologies for use in clinical trials investigating novel therapies for the treatment of this rare disease. This summary of proceedings highlights outcomes of the Statistical Group and provides a pathway for evaluation of composite endpoints. These efforts also provide a roadmap for developers of new therapies to consider application of MDEs for other rare diseases.

MDEs potentially provide higher power to detect treatment effects in situations when both the disease impacts and the drug benefits are heterogeneous, including settings in which no single endpoint is relevant for all patients who could benefit from therapy, due to sizable subpopulations who are either not at risk of worsening in that single endpoint or are not impaired at baseline and thus do not have room to improve. Sponsors must account for the important risks and limitations inherent to MDEs, such as inadequate relative weighting of disease domains or component endpoints (e.g., treating components as equally important when their importance differs in reality). When an MDE incorporates components with very different clinical importance, the clinical meaningfulness of the results will be an issue. Interpretation may be confounded if the components do not trend in the same direction. Power can also be affected if some components in a MDE have small or no treatment effect.

For this initial effort, strategic approaches to identify potential components were considered in the context of treatment with plasma cell reduction agents. By targeting the underlying disease mechanism with plasma-cell directed therapy, multi-domain impacts on end organs inherently lend to an MDE approach. The Statistical Group recognized the relative utility of the various endpoint components might be different in the context of a trial evaluating an anti-amyloid therapy (i.e., a therapy targeting the removal of amyloid fibril deposits). Based on the intended context of use for a particular investigational product, sponsors should also seek to engage regulatory authorities to prospectively consider the acceptability of an MDE for use in a given development program. It is also important to consider specific domains of interest (e.g., disease-related symptoms and proximal impacts of the disease on functioning) that are most likely to demonstrate change in a clinical trial. COA-based measures may be incorporated appropriately in the endpoint hierarchy, considering the suitability and validity of the instrument for AL amyloidosis and the domains represented [41].

**Figure 3:**
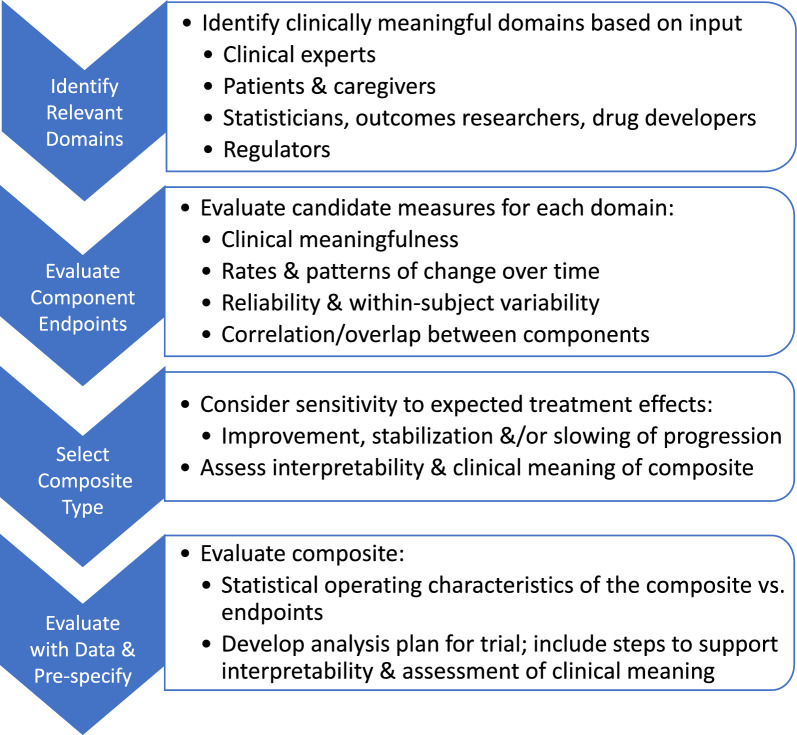
Overview of proposed pathway for development and evaluation of composite endpoints. Regulatory input is recommended early and often in the development process.

With input from multiple stakeholders, including patients, clinicians, statisticians, drug developers, and regulatory agency representatives, the Statistical Group has summarized a proposed process to enable drug developers and engage regulators to make evidence-based decisions on endpoints. The next steps on the path to development and evaluation of MDEs for AL amyloidosis are to evaluate the performance of the candidate component endpoints that have been prioritized for each domain (Fig. 3). To this end, the Statistical Group will prioritize analysis of available natural history and clinical trial datasets to (1) evaluate NT-proBNP and hematologic complete response as surrogate endpoints for survival or clinical organ progression, and (2) evaluate 6MWD as a functional endpoint in an AL amyloidosis population.

## Conclusion

Given the systemic, heterogeneous nature of AL amyloidosis, the Amyloidosis Forum is working toward identifying appropriate endpoints and analytical methodologies for use in clinical trials investigating novel therapies. MDEs represent a promising analysis approach to enable earlier detection of therapeutic effect by combining signals across domains and may result in increased power (i.e., signal to noise) relative to any single component measure. When developed using a defined pathway, an MDE will be clinically meaningful, interpretable, and harness the principles of patient-focused drug development for a given disease with heterogeneous population and myriad clinical impacts. While our collaboration has focused on AL Amyloidosis, the Working Group structure and process—including domain-specific clinical experts, regulators, outcomes researchers, statisticians, drug developers and patients—could be applied in the development of endpoints for other multi-system diseases.
